# Radiomics-Based Inter-Lesion Relation Network to Describe [^18^F]FMCH PET/CT Imaging Phenotypes in Prostate Cancer

**DOI:** 10.3390/cancers15030823

**Published:** 2023-01-29

**Authors:** Lara Cavinato, Martina Sollini, Alessandra Ragni, Francesco Bartoli, Roberta Zanca, Francesco Pasqualetti, Andrea Marciano, Francesca Ieva, Paola Anna Erba

**Affiliations:** 1 MOX—Modeling and Scientific Computing, Department of Mathematics, Politecnico di Milano, p.zza Leonardo da Vinci 32, 20133 Milan, Italy; 2 Department of Biomedical Sciences, Humanitas University, Via R. Levi Montalcini 4, 20090 Pieve Emanuele, Italy; 3 Nuclear Medicine, Humanitas Research Hospital, Via Manzoni 56, 20089 Rozzano, Italy; 4 Nuclear Medicine, Department of Translational Research and Advanced Technology in Medicine and Surgery, Pisa University Hospital, University of Pisa, Via Roma 67, 56123 Pisa, Italy; 5 Radiation Oncology, Pisa University Hospital, Via Roma 67, 56123 Pisa, Italy; 6 Health Data Science Center, Human Technopole, viale Rita Levi Montalcini, 20157 Milano, Italy; 7 Medical Imaging Center, University Medical Center Groningen, University of Groningen, 9700 AB Groningen, The Netherlands

**Keywords:** prostate cancer, radiomics, PET, imaging-derived biomarkers, tumor heterogeneity

## Abstract

**Simple Summary:**

Advanced image analysis, specifically radiomics, has been recognized as a potential source of biomarkers for cancers. However, there are challenges to its application in the clinic, such as proper description of diseases where multiple lesions coexist. In this study, we aimed to characterize the intra-tumor heterogeneity of metastatic prostate cancer using an innovative approach. This approach consisted of a transformation method to build a radiomic profile of lesions extracted from [^18^F]FMCH PET/CT images, a qualitative assessment of intra-tumor heterogeneity of patients, and a quantitative representation of the intra-tumor heterogeneity of patients in terms of the relationship between their lesions’ profiles. We found that metastatic prostate cancer patients had lesions with different radiomic profiles that exhibited intra-tumor radiomic heterogeneity and that the presence of many radiomic profiles within the same patient impacted the outcome.

**Abstract:**

Advanced image analysis, including radiomics, has recently acquired recognition as a source of biomarkers, although there are some technical and methodological challenges to face for its application in the clinic. Among others, proper phenotyping of metastatic or systemic disease where multiple lesions coexist is an issue, since each lesion contributes to characterization of the disease. Therefore, the radiomic profile of each lesion should be modeled into a more complex architecture able to reproduce each “unit” (lesion) as a part of the “entire” (patient). This work aimed to characterize intra-tumor heterogeneity underpinning metastatic prostate cancer using an exhaustive innovative approach which consist of a i) feature transformation method to build an agnostic (i.e., irrespective of pre-existence knowledge, experience, and expertise) radiomic profile of lesions extracted from [^18^F]FMCH PET/CT, ii) qualitative assessment of intra-tumor heterogeneity of patients, iii) quantitative representation of the intra-tumor heterogeneity of patients in terms of the relationship between their lesions’ profiles, to be associated with prognostic factors. We confirmed that metastatic prostate cancer patients encompassed lesions with different radiomic profiles that exhibited intra-tumor radiomic heterogeneity and that the presence of many radiomic profiles within the same patient impacted the outcome.

## 1. Introduction

In the era of personalized treatment an increasing focus has arisen on biomarkers to identify a patient’s specific characteristics and assist clinicians in their decision making. Parallelly to serum and molecular markers, advanced image analysis has recently acquired recognition as source of biomarkers. Whole-body assessment that attempts to evaluate multiple lesions (when present) at the same time, repeatability and cost-effectively—as is performed using conventional imaging—are among the pros of advanced image analysis over other approaches [1,2]. Specifically, quantitative features extracted from imaging through mathematical approaches (i.e., radiomics) are capable of categorizing tumors into different (imaging) phenotypes. Nonetheless, some technical and methodological challenges are faced with the application of radiomics clinically [3,4]. To successfully implement radiomics in the real clinical world, sustainable, rigorous, and robust research plans are needed for study design, model development, training, and testing. Although these limitations have been extensively acknowledged in recent years, as clearly stated by reviews, editorials, expert opinions, and position papers, the majority of radiomic studies lack adequate sample size, rigorous methodology, and appropriate methods for statistics and data analysis. As a result, no strong evidence about the role of radiomics has been provided [5], nor understandable answers have been given to explain their clinical significance [2,6,7]. Moreover, many studies showed a scarce robustness for radiomics features and proved that preprocessing data harmonization may have a positive impact, simplifying multi-institutional collaboration for large-scale analytics [4,8,9]. Indeed, radiomic features are affected by many pre- and postprocessing factors including the scanner, acquisition protocol, segmentation method, software, and parameters setting for extraction [4,5,6]. Proper methods for feature selection and dimensionality reduction should be employed to limit redundancy and remove uninformative data from the dataset [4,5,6]. Moreover, well-designed trials and multidisciplinarity are also crucial factors in radiomic studies to informatively contribute to science [10,11]. 

Predictive and prognostic radiomic models have been extensively proposed for primary tumors, whereas proper phenotyping of metastatic or systemic disease where multiple lesions coexist is still missing [12,13]. We recently demonstrated that proper modelling of radiomic data provides crucial information on lesion heterogeneity in patients with recurrent prostate cancer (PCa), reflecting the presence of different cellular clusters within each patient. Indeed, biochemical recurrence will occur in 20–40% of PCa patients after radical prostatectomy and in 30–50% of cases after radiotherapy within ten years [14] suggesting that other factors—in addition to those commonly used in clinical practice (e.g., Gleason score)—play a role in disease progression and prognosis. We previously showed that [^18^F]FMCH PET/CT lesion heterogeneity differed in patients with limited tumor burden (i.e., oligometastatic) as compared with patients with more advanced disease. Such heterogeneity significantly decreased when considering only lesions within the same organ with respect to all the lesions and when focusing on metabolically similar lesions featuring comparable SUV_max values [13]. From this experience, we learnt that to maximize the benefit of subpopulation-specific risk stratification, we have to move beyond single lesion assessment. Inter-lesion description is needed to build up an “object” representing the inter-lesion relation network, exhaustively representing the disease within the patient. Nonetheless, each lesion (and its heterogeneity) contributes to characterize the disease and, consequently, the patient. Therefore, each lesion’s radiomic profile should be modeled into a more complex architecture able to reproduce each “unit” (lesion) as a part of the “entire” (patient). 

**Figure 1:**
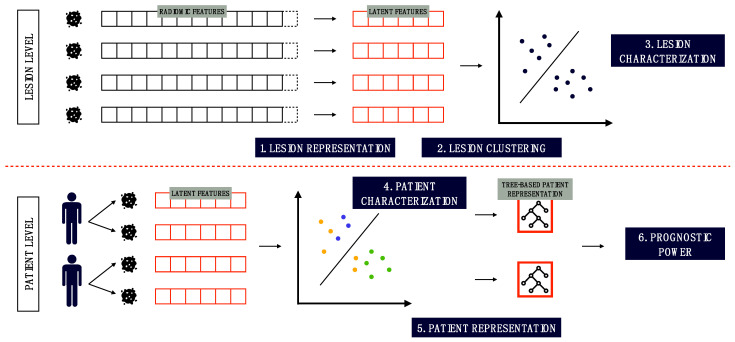
Flowchart of the analyses: at the lesion level, lesions are (1) represented through an agnostic dimensionality reduction of radiomic vectors and (2) clustered in groups, which are further analyzed with clinical variables (3). At the patient level, patients are qualitatively described in terms of intra-patient heterogeneity (4), represented through trees (5), and quantitively analyzed with prognostic purposes (6).

This work aimed to provide three different contributions to access and potentially exploit cancer imaging phenotypes in PCa. With reference to Figure 1, we first perform a view-wise radiomic feature transformation model to build an agnostic (i.e., irrespective of pre-existence knowledge, experience, and expertise) radiomic profile of lesions from their [^18^F]FMCH PET/CT assessment (1). Then, we cluster the agnostic radiomic profiles of lesions according to their similarity (2), and we exploit these clusters to qualitatively characterize the similarity of lesions (3) and intra-tumor heterogeneity of patients (4). Furthermore, we quantitively represent patients’ disease phenotype based on the evolutionary relationship between their lesions’ profiles (5). To do this, tree-shaped objects were proposed for patient representation. The clinical relevance of the tree-shaped patient representation was assessed in terms of association with clinical prognostic factors and patient outcome (6).

## 2. Materials and Methods

### 2.1. Study Design and Patient Selection

The cohort of the present analysis consisted of 55 male patients (mean age 73 ± 7 years; median age 75 years, range 58–85) with biochemical failure after first-line curative treatments for PCa, exhibiting at least two lesions showing uptake of [^18^F]FMCH at PET/CT. All the scans were performed at the Nuclear Medicine Department of the Azienda Ospedaliero Universitaria Pisana, using an integrated PET/CT system General Electric Discovery 710 (General Electric Healthcare, Waukesha, WI, USA) as previously detailed [13]. A total of 333 lesions were I, including 149 lymph node (68 regional and 81 distant) metastases and 221 bone lesions. The median number of lesions for each patient was 5. Demographic and clinical patient data including age, Gleason score (GS) at diagnosis, prostate specific antigen (PSA) level at the time of [^18^F]FMCH PET/CT, primary treatment, and androgen deprivation therapy (ADT; if yes: ongoing or discontinued) were collected. Baseline patient characteristics are summarized in Table 1.

### 2.2. Image Analysis

Image analysis and radiomic feature extraction have been previously detailed [13]. Briefly, the LIFEx software (http://www.lifexsoft.org, accessed on 1 January 2020 [15]) was used to semi-automatically segment all patients’ lesions and obtain, for each of them, five conventional parameters related to the standardized uptake value (SUV) and 37 radiomic features. The 37 radiomic features were grouped, by methodological construction and software output, into six different semantic groups (HISTOGRAM, SHAPE, GLCM, GLRLM, NGLDM, and GLZLM).

### 2.3. Data Analysis and Statistics

Frequency tables and descriptive statistics were used to summarize the study’s population. According to the contributions of this work, analyses consisted of three steps, i.e., (1) the construction of lesions textural profile, (2) a qualitative and (3) a quantitative assessment of intra-tumor heterogeneity according to the tree-based patient representation. Each step is validated by means of a comprehensive characterization of the clinical variables as detailed in the results section following the present one. Moreover, the quantification of intra-tumor heterogeneity via tree-based representation was further tested for its prognostic power through survival analysis.

#### 2.3.1. Lesion Textural Profile

Data depth was applied for radiomic data representation in this analysis. A data depth is a way of measuring how deep (central) a given observation is with respect to the peer observations. It can be thought of as a multivariate generalization of boxplots. Indeed, when analyzing a boxplot of a variable we are assessing the relative position of each sample in the context of the other samples’ distribution: samples close to the median values are typical samples and are more probable to be found in the distribution. Contrary to this, the greater the distance from the central values the less typical they are, indicating they are rarely observed within the phenomenon under analysis (outliers). The same approach can be extended to observations considering a higher number of variables, and the measure of the centrality/outwardness of data points is called the depth of the points. Indeed, depths allow ranking of objects characterized by a number of features d>1. Several definitions of data depth—such as Mahalanobis depth, Halfspace (or Tukey) depth, projection depth, and spatial depth—are available in the literature [16,17,18] and their properties were reviewed for radiomic data representation in our context. Exploratory analyses are described in Supplementary Figure S1. 

Since radiomic features are usually extracted from different high-throughput methods, they provide a multi-view textural description of lesions. Accordingly, they are grouped into different matrices or categories. Specifically, in this study radiomic features were divided into six semantic groups, namely histogram-derived variables, shape-derived variables, GLCM-derived variables, GLRLM-derived variables, NGLDM-derived variables, and GLZLM-derived variables. Collinearity and redundancy among features within these semantic groups are known to be strong, so for the depth analysis we maintained the same semantic structure. We computed depth measures separately for each group of variables, obtaining a depth value for each semantic radiomic group. To quantify the agreement of information provided by the views, a ranking agreement analysis was performed with R package SuperRanker [19]. The procedures are discussed in Supplementary Figure S2. Conventional, i.e., SUV-related, features of uptake values, not belonging to any of the radiomic groups, were excluded from the depth computation and left for results interpretation. 

#### 2.3.2. Qualitative Assessment of Intra-Tumor Heterogeneity

Having each lesion described by a reduced and comparable vector of depth measures (i.e., lesion’s radiomic profile), it is now possible to assess the radiomic profiles of different lesions and to characterize lesions with similar characteristics. Specifically, lesions’ radiomic profiles were clustered according to unsupervised minibatch K-means clustering [20]. The dataset of lesions (*n* = 333, *p* = 6) was in fed into the algorithm and *k* was selected among a range of values through an exhaustive grid search so as to meet intra-cluster homogeneity. As a result, each lesion was tagged with a membership class, regardless the patient it belonged to. In this way, groups of similar lesions were identified: lesions falling in the same group exhibited a homogeneous profile, whereas lesions in different groups were considered to display a heterogeneous profile. Accordingly, it is possible to phenotype different groups (types) of lesions, highlighting different pattern templates. The mean radiomic profile was computed for every class, so as to represent the pattern templates and compare the detected phenotypes. Each pattern template describes the lesions’ radiomic phenotypes as assessed and characterized using the lesions’ clinical and biological characteristics. Specifically, the standard uptake values (SUV_max) were used to describe differences in lesion intensity, the total lesion activity (TLA) was used to describe differences in lesion volume, and GLCM entropy was used to describe differences in lesion heterogeneity [21]. Moreover, lesions’ sites (locoregional lymph nodes, distant lymph-nodes, and skeletal) were taken into account to assess tumor spreading. To test the differences, non-parametric t-tests were used for numerical features, whereas the chi-squared test was used for categorical features. Additionally, since patients in this study presented multiple sites of disease, we divided the patients in two different groups:

- patients exhibiting lesions with homogeneous radiomic phenotypes—i.e., their lesions fell into the same group of lesions—were labelled as patients with homogenous disease;
- patients featuring lesions with heterogeneous radiomic phenotypes—i.e., their lesions fell into more than one group of lesions—were labelled as patients with heterogeneous disease.

In this way, the number of different radiomic profiles within a patient expresses the extent of heterogeneity of their disease. The two groups of patients were characterized throughout a clinical investigation. Specifically, the personal and clinical characteristics were compared in the two groups, including the Gleason Score, the oligo-/multi-metastatic status, the number of lesions, the type of upfront PCa treatment (initial therapy), the status of androgen deprivation therapy (ongoing therapy), the prostate specific antigen (PSA) value, the type of treatment, and the response to therapy. To test the differences, non-parametric t-tests were used for numerical features, whereas the chi-squared test was used for categorical features. *P*-values below 0.05 were considered significant, however, values below 0.1 were included for discussion as well.

#### 2.3.3. Quantitative Assessment of Intra-Tumor Heterogeneity

**Figure 2:**
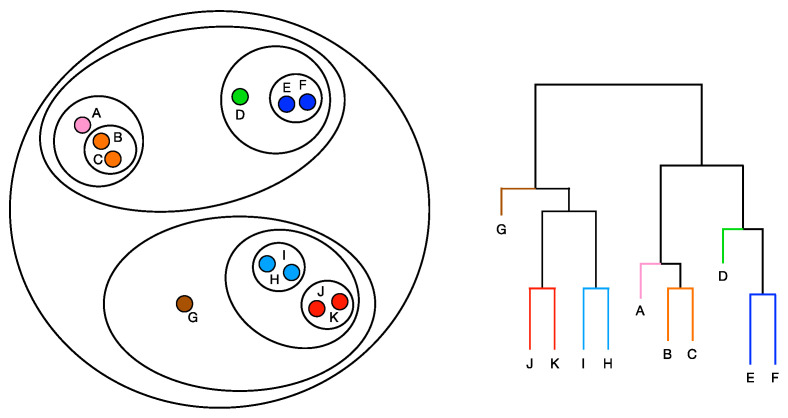
Procedure of hierarchical clustering techniques: the clustering begins with treating each lesion as a separate cluster and iteratively aggregates similar lesions, that is lesions with short pairwise distance, to merge groups of lesions in a single cluster. The output of the procedure is called a dendrogram.

As a step forward towards the qualitative assessment of patients’ disease heterogeneity, we tuned a quantitative pipeline. Specifically, we went beyond the qualitative subtyping of lesions coexisting in the radiomic phenotypes of patients. We leveraged the lesions’ radiomic profiles to build an insightful patient representation describing the homogeneity/heterogeneity extent among the patients’ tumor lesions, so as to assess the patients’ disease as a whole. The similarity between two peer lesions’ profiles can be measured by their pairwise Euclidean distance. Accordingly, the evolutionary and statistical relationship among the lesions in a patient was represented by a hierarchical clustering dendrogram with Euclidean distance and complete linkage. Specifically, for each patient, the square matrix of the pairwise Euclidean distances among their lesions was computed and fed into the clustering algorithm. The lesions were represented by the six-dimensional vectors of depths describing the relative position of the lesion according to the six radiomics semantic groups. Generally speaking, an agglomerative hierarchical clustering algorithm begins with treating each lesion as a separate cluster and iteratively aggregates similar lesions, that is lesions with short pairwise distance, to merge groups of lesions in a single cluster. The process is illustrated in Figure 2.

The tree-shaped output of the algorithm is called a dendrogram and shows the hierarchical relationship between peer observations. Therefore, each patient is now represented by a dendrogram, where the leaves correspond to the lesions and the lengths of the branches reflect the mutual similarity relationship of the radiomic profiles expressed by the lesions. Lesions that are close to each other are very similar, thus exhibiting a similar radiomic profile, whereas distant leaves reflect heterogeneous lesions. For a posteriori evaluation of the reliability of such a representation with clinical patient-specific features, we extracted tree-derived descriptors. Accordingly, tree-derived features included the number of lesions, the sum of the tree branch lengths, dispersion among lesions, and number of different phenotypes. They were computed and correlated with patients’ clinical variables, such as Gleason Score, prostate specific antigen levels, the oligo-/multi-metastatic status, the type of treatment, and the response to therapy. The number of lesions phenotype was the number of different, i.e., independent, radiomic patterns expressed by a patient. In a dendrogram, this number was obtained by computing the best clustering of the patient’s lesions according to the evaluation of a similarity measures, i.e., the Silhouette index [22], Davies–Bouldin index [23], and Calinski–Harabasz index [24]. The lesions’ similarities in the reduced radiomic space was doublechecked with a ranking aggregation algorithm, as implemented in R package RankAggreg [25]. Supplementary Figure S3 graphically describes the process.

#### 2.3.4. Perspective Modeling

To further assess the associations between tree-derived descriptors and clinical patient information, we tested the prognostic and predictive power of patient representation. Tree-derived features, as listed above, were fed into Cox proportional hazard models in both univariate and multivariate fashion to predict disease-free survival. Significance of their power was assessed with *p*-values of log rank tests and final model performance was evaluated in terms of concordance index.

## 3. Results

As for data analyses, results consisted of the description of three sequential parts, i.e., (1) the lesion textural profile and the (2) qualitative and (3) quantitative assessment of intra-tumor heterogeneity according to the tree-based patient representation.

### 3.1. Lesion Textural Profile

**Figure 3:**
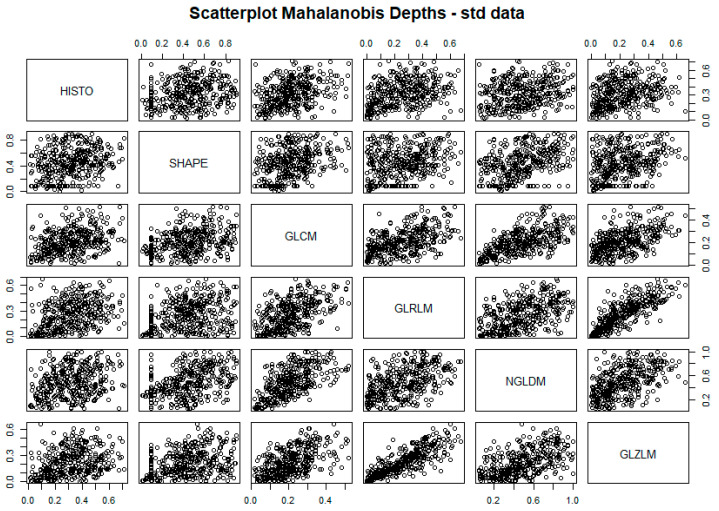
The pairwise scatterplots of radiomic groups’ depth measures. Correlation is low for each pair of radiomic groups but with higher values for GLZLM and GLRLM.

Mahalanobis depth definition was chosen as a result of the visual intra-view correlation inspection (Supplementary Figure S1) and ranking agreement analysis (Supplementary Figure S2). Data visualization of textural features’ dimensionality reduction is shown in Figure 3. 

**Figure 4:**
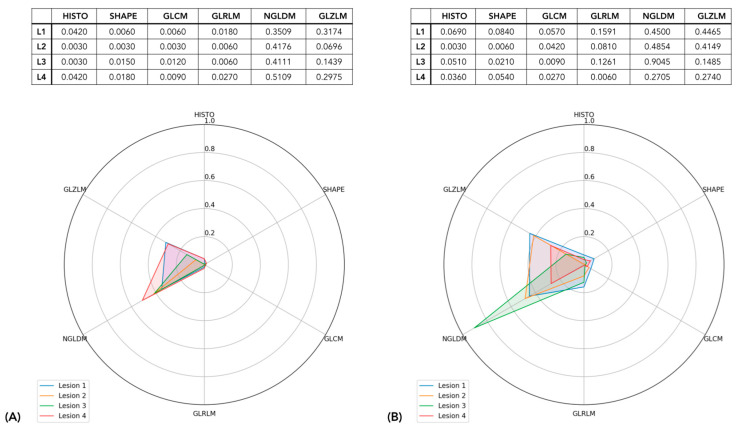
The lesion profiles of two patients. Each lesion is represented by the 6 depth measures related to each radiomic view. Depth values as listed in the tables are graphically represented in spider plots. In (**A**), the patient (#17) exhibited only one radiomic pattern; in (**B**), the patient (#31) showed two different shapes in the lesion radiomic profile.

Every lesion was represented by six depth values—one per radiomic semantic group, i.e., view—describing its centrality with respect to the peer lesions’ distributions. The correlation between the views was assessed by plotting the distribution of depth values for each lesion computed according to one view versus the ones computed according to another view. As shown in the figure, the correlation between the views was never higher than 0.5, except for the correlation between GLRLM and GLZLM, which raised to 0.8, suggesting the independent information content provided by each view. The exemplification of the lesions’ radiomic profiles can thus be visualized in Figure 4, where sample lesions are shown, grouped by the patient they belong. 

Specifically, we presented the lesion radiomic profiles of two patients (#17 and #31) where depth values of the six radiomic views are listed and plotted in the corresponding spider plots. Every axis of the spider plots corresponds to a specific view, as highlighted by the labels, and each line graphically displays the lesion’s radiomic profile in terms of depth measures. Accordingly, the shape can be intended as the textural phenotype or signature, providing an agnostic description of its textural phenotype with respect to other lesions. In patient #17, all four lesions appeared to be very similar in terms of their radiomic profiles as their shapes match a similar template. HISTO, SHAPE, GLCM, and GLRLM presented particularly low depth values, whereas NGLDM and GLZLM assumed higher depth values. In patient #31, the lesions differed since lesions 1 and 2 exhibited almost identical profiles (with enhanced values of NGLDM and GLZLM), lesion 4 followed their shape yet with a smaller area, whereas lesion 3 behaved as an outlier, showing very low depth values for HISTO, SHAPE, GLCM, GLRLM, and GLZLM and displaying a spike of centrality towards NGLDM. Accordingly, patient #17 exhibited a more homogeneous disease with lesions entailing the same radiomic description, whereas the disease of patient #31 was radiomically heterogenous. Starting from this analysis, illustrated through these two examples, in the following, we aimed to progressively characterize this heterogeneity with both qualitative and quantitative methods.

### 3.2. Qualitative Assessment of Intra-Tumor Heterogeneity

**Figure 5:**
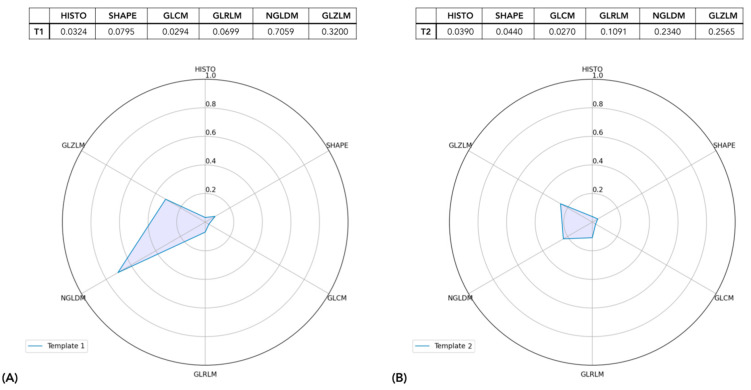
Mean radiomic profile of the two classes of lesions. In (**A**) the Class 1 radiomic template and in (**B**) the Class 2 radiomic template are displayed. The mean depth measure values are listed in the tables and graphically represented in the spider plots.

As stated above, Figure 4 shows an example of the results of the characterization of the lesion profiles of two patients. The profile of each lesion of a patient is highlighted with a different color. The visualization of radiomic profiles as described above allows a very rapid and visual comparison of intra-patient lesion heterogeneity. In fact, lesions exhibiting similar shapes presented mutually homogeneous radiomic patterns whereas lesions displaying mismatched shapes entailed mutually heterogeneous radiomic patterns. Intuitively, the more the lesions of a patient display a similar pattern, the more homogeneous the disease, and vice versa, if a patient presented many different pattern templates, they are described as exhibiting a heterogeneous disease, with heterogeneity increasing according to the number of coexisting templates, i.e., radiomic phenotypes. According to minibatch K-mean clustering, two groups of lesions were identified, independently to the patients they belong. Figure 5 shows the radiomic templates of the two clusters. Cluster 1 was characterized by a very deep value for NGLDM followed by a relatively deep value for GLZLM, as for the presence of typical zone homogeneity and voxel contrast.

In contrast, the HISTO, SHAPE, GLCM, and GLRLM components were less deep, being outliers compared with the lesion population. Cluster 2 exhibited a pretty regular shape, with persistent accentuated NGLDM and GLZLM depth values, yet these were not significantly different from the other components. The site of disease did not play a significant role in scoring disease heterogeneity, as lesions in either class were not more often located at any particular site of disease (regional lymph nodes, distant lymph nodes, and bone metastases; χ2 test, *p*-value = 0.4202). In contrast, the value of SUV_max, TLA, site, and GLCM_Entropy, considered surrogated markers of tumor aggressiveness [26], proliferative activities [27], and a measure of intra-lesion heterogeneity [28,29], respectively, were significantly different in the two clusters (Table 2).

This underlines the ability of the depth measures to depict different biological features sustaining the tumor. In particular, the Class 1 cluster hosted the majority of lesions with lower values of SUV_max, whereas the majority of lesions with higher SUV_max values fell into Class 2, as highlighted from SUV_max distribution indexes (*t*-test, *p*-value = 0.0187). Therefore, Class 1 featured lesions with a “low” proliferative rate; in Class 2, the lesions were the ones with increased proliferation. Coherently, Class 1’s lesions presented lower TLA (mL) values with respect to the Class 2 ones, as the lesions’ activity distribution parameters were higher in the second group (*t*-test, *p*-value < 0.001), characterized by a heavier right tale (Q3 parameter). GLCM_Entropy of lesions was significantly different as well, although at a higher significance level. The heterogeneity of lesions was slightly higher in Class 2 than in Class 1, revealing a relationship between heterogeneity and proliferation (*t*-test, *p*-value = 0.0517). Further to the lesions’ characterizations, we qualitatively divided patients according to the homogeneity of their lesions’ clusters. In fact, the number of lesion clusters in a patient is identified as a proxy for revealing patients’ intratumor heterogeneity. A total of 39/55 patients exhibited lesions featuring different radiomic profiles, since their lesions belonged to different clusters; thus, they were referred to as heterogeneous. The lesions of the remaining 16/55 patients presented a unique radiomic pattern, thus falling in one cluster only; therefore, they were referred to as homogeneous. The results are displayed in Table 3. 

Although no significant difference was found in PSA levels and Gleason score (GS) or Gleason category (i.e., GS ≤ 7 vs. GS > 7) between these two groups of patients, the number of lesions and oligo/multi-metastatic status clinical cutoffs were coherently correlated with the stratification. Indeed, more homogeneous patients presented a lower number of lesions (*t*-test, *p*-value = 0.0001) and a lower total disease volume (*t*-test, *p*-value = 0.0651) than the heterogeneous group. This latter group exhibited a higher proportion of oligometastatic disease (χ^2^ test, *p*-value < 0.0001 with a cutoff of 3 lesions; *p*-value = 0.0004 with a cutoff of 5 lesions; *p*-value = 0.0001 with cutoffs at both 3 and 5 lesions). Furthermore, the type of treatments and patients’ response to therapy did not significantly differ in these two groups of patients, suggesting a lack of current decision-making clinical parameters in assessing a patient’s disease heterogeneity. Accordingly, this supported the necessity of a more exhaustive and quantitative patient representation.

### 3.3. Quantitative Assessment of Intra-Tumor Heterogeneity

**Figure 6:**
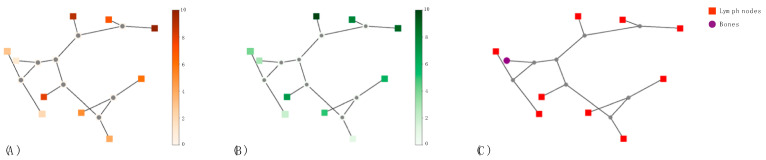
A patient tree-based representation. Lesions are colored according to biological characterization, namely (**A**) their standard uptake values (SUV_max), (**B**) total lesion activity, TLA, and (**C**) tissue of their sites, such as proximal and distant lymph nodes (squares) or skeleton (circles).

Since heterogeneity differences among patients are appreciable according to the different number of coexisting phenotypes within their disease, hierarchical clustering was used to represent such a patient’s heterogeneity. Furthermore, hierarchical clustering allows us to exploit the hierarchical nature of the lesions in a patient. One dendrogram per patient was built and the dendrogram-related information was investigated to unveil a prognostic characterization. Indeed, the similarity among lesions’ radiomic profiles reveled a biological counterpart. Figure 6 shows a patient tree-based representation. Single lesions’ biological characteristics such as standard uptake values (SUV_max), total lesion activity (TLA), and sites of disease metastases (i.e., loco-regional lymph nodes, distant lymph nodes, or skeleton) are described using a color code. 

Of interest, close lesions and distant lesions presented different biological characterization, as highlighted by the specific color encoding. Table 4 showed the discrimination power of number of phenotypes, number of lesions, sum of tree branch lengths, and lesions’ radiomic dispersions according to type of therapy, number of treatments, oligo/multi-metastatic status, GS, and PSA. 

The time and response to therapy were assessed in a perspective way. The number of phenotypes, regardless of the similarity index used to compute it, was significantly associated with PSA levels, the number of lesions and the total tumor volume, Gleason category, oligo/multi-metastatic status (with cutoffs of 3, 5, and both 3 and 5), and radiotherapy administration (Yes/No). The dispersion of lesions and sum of branch lengths were discriminative with respect to the number of lesions, Gleason category, oligo/multi-metastatic status (with cutoffs of 3, 5, and both 3 and 5) and radiotherapy. Treatment was not correlated with tree-based representation descriptors: type of upfront PCa treatment, ongoing androgen deprivation therapy, and combination of treatments were found independent with respect to phenotype counts and lesion dispersion. 

### 3.4. Perspective Modelling

**Figure 7:**
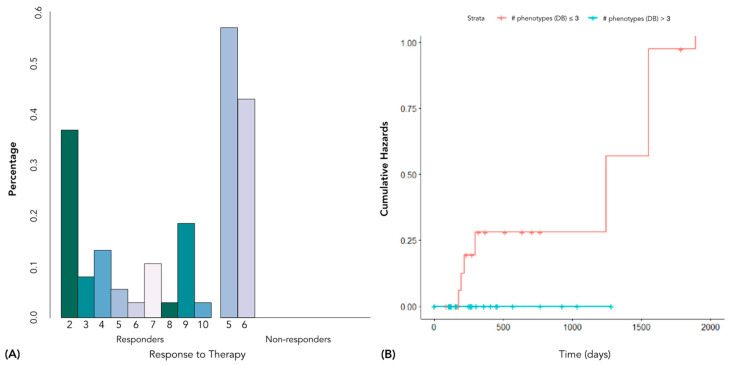
Best cutoff value for Davies–Bouldin-based number of phenotypes to discriminate between responder and not responder patients: (**A**) barplot of patient proportion; (**B**) stratified Kaplan–Meier.

For univariate Cox models, dispersion, number of lesions, sum of branch lengths, number of phenotypes according to Silhouette and Calinski–Harabasz indexes, and PSA were not significant (*p*-values = 0.438, 0.679, 0.432, 0.549, and 0.48, respectively). The number of phenotypes according to Davies–Bouldin and combined therapy were significant (*p*-values = 0.04146 and 0.0233, respectively). As shown in Figure 7, the best cutoff value for the Davies–Bouldin-based number of phenotypes to discriminate between responder and not responder patients was 3 (*p*-value = 0.05). 

**Figure 8:**
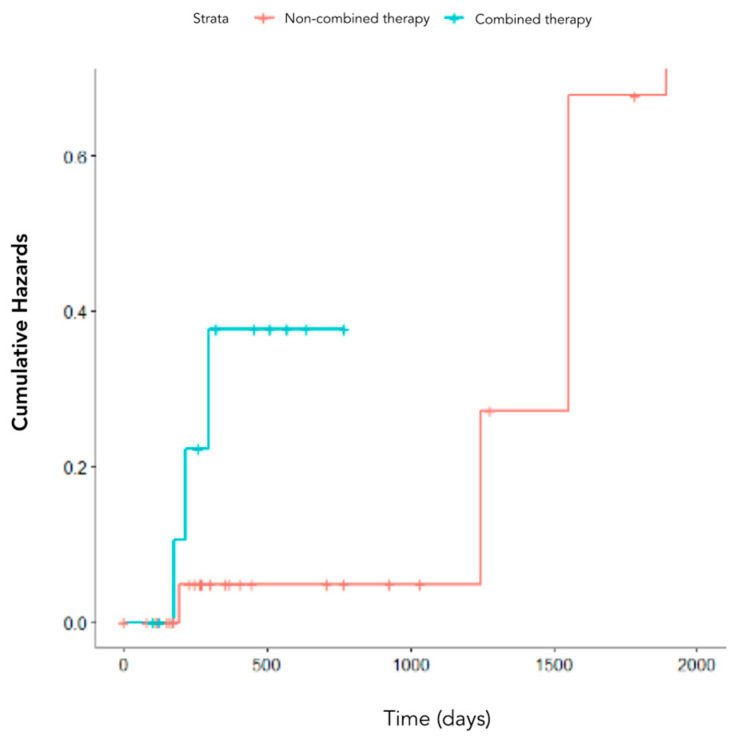
Kaplan–Meier cumulative hazards of patients stratified according to the combination of treatments, i.e., patients who underwent one (blue curve) or more than one therapy type (red curve).

Figure 8 shows the Kaplan–Meier survival curves of patients stratified according to combination of treatments, i.e., patients who underwent one or more than one therapy type.

**Figure 9:**
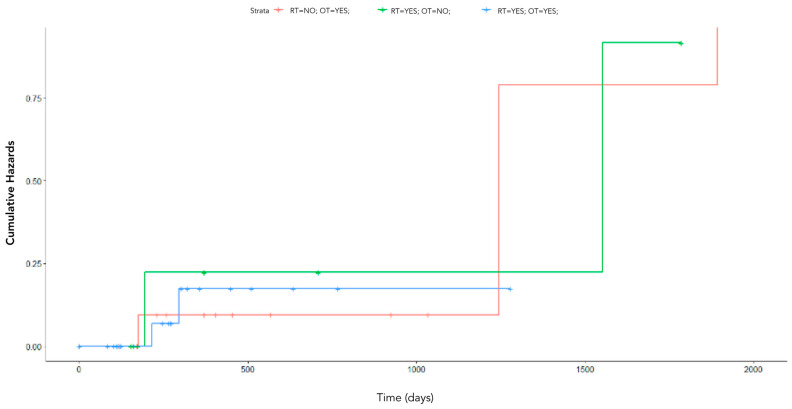
Kaplan–Meier cumulative hazards of patients stratified according to type of treatment, i.e., patients who underwent hormonotherapy (red curve), radiotherapy (green curve), or both (blue curve).

The type of therapy was not significant (*p*-value = 0.9), as highlighted in Figure 9.

The best multivariate model was the one that contained dispersion, number of lesions, sum of branch lengths, Davies–Bouldin-based number of phenotypes, and combined therapy, resulting in a 0.86 concordance with a *p*-value of 0.09 using the log rank test.

## 4. Discussion

In this manuscript we describe a novel statistical approach for analyzing radiomic data and accordingly describe, represent, and quantify disease heterogeneity in patients with metastatic PCa. Radiomic features have been introduced as imaging biomarkers as they represent an index of the degree of tumor heterogeneity [1,30]. However, radiomic features have the limitations of instability and scarce robustness due to the use of different scanners, acquisition, and post-processing settings [4,6]. Therefore, a normalization strategy to make them agnostic and robust is needed. Further, redundancy and collinearity have to be tacked in order to produce insightful models and extract useful knowledge. The statistical approach for agnostic dimensionality reduction we propose in this work takes advantage of data variability in order to normalize features’ contributions in a descriptive or perspective model. Pertinently, the measure of data depth provides a center-outward ordering of points in a set and leads to a non-parametric multivariate statistical description of data, in which no distributional assumptions are needed. Among depth definitions, we preferred the use of Mahalanobis depth because with this model the computed distribution appeared more dispersed about its center of symmetry than distributions stemming from other depths, leading to clearer results (Supplementary Figure S1). The depth computation resulted in a dimensionality reduction strategy where the 37 radiomic features describing the lesions were summarized in six agnostic measures. Accordingly, the six depth values formed an agnostic multi-view lesion profile to be used for the descriptive and prognostic modeling of intra-tumor heterogeneity. Notably, the correlation found among the six semantic groups was negligible (less than 0.5) except for GLRLM and GLZLM (0.8). This was expected as the length and zones are very similar structures and coherently produce correlated results, whereas other groups’ features independently capture different aspects of texture [31,32]. According to the depth-based feature transformation, we presented agnostic radiomic profiles of lesions grouped by the patient they belonged to (Figure 4). Based on such profiling, we can depict intra-tumoral heterogeneity in some patients that are characterized by the contemporary presence of "typical" and "atypical" lesion radiomic profiles. In other patients, only "typical" lesions were observed. Coherently, disease heterogeneity was found to be independent from disease site and burden as well as treatment. However, it was proven to reflect the number of coexisting phenotypes within a patient. Moreover, we observed a stronger association between the number of lesions and disease heterogeneity rather than the volume of lesions and disease heterogeneity, although both indicators are currently considered as prognostic factors. This becomes critical in a population as one in the current work existed in more than two thirds of heterogeneous clusters of lesions. Interestingly, radiomic features provided information coherent with SUV-derived parameters. When multiple lesions were present, the contribution of all of them—regardless of the hosting tissue and number—to the tumor biology and ultimately the outcome is crucial, confirming our previous findings [12,13]. Beside imaging-related information, clinical variables describing the status/severeness of the diseases coherently correlated with heterogeneity measures, whereas therapy did not denote any specific correspondence with tumor heterogeneity; although patients who underwent more than one treatment had a worse outcome. In fact, different regimens of therapy are typically performed in recurrent or more aggressive tumors. Of note, radiotherapy implementation seemed to follow a latent heterogeneity assessment. We can speculate that radiotherapy may select some cellular clones, impacting on tumor biology, heterogeneity, and ultimately the outcome. Patients presenting “typical” and/or “atypical” phenotypes presented different outcomes. Specifically, in our series heterogeneity directly impacted on outcome, with having more than three phenotypes (high heterogeneity) negatively associated with outcome (Figure 7). The proposed patient representation frames a quantitative approach that was needed as a step forward with respect to the qualitative assessment of heterogeneity. The hierarchical clustering devises a comprehensive and unique object able to summarize the statistical units (lesions) by their grouping policy (patients). Upon these objects, many studies are available to perform inference, classification, clustering, and prediction [33,34,35]. In this work, we proved how tree-based representations insightfully entail tumor heterogeneity information in the context of PCa. Such information has been revealed to be significant in predicting the response to therapy beyond clinical assessment. In fact, tree-derived indicators describing the morphology and the shape of the tree structures were used to prove their prognostic power through survival analyses. Most of the current clinical-based biomarkers struggle in relating tumor heterogeneity with cancer progression, whereas our approach showed promising results. Furthermore, the tree objects prevent the predictive statistical analyses from being hampered by radiomic limitation, above all inter-scanner variability. Specifically, the tree-based representation disentangles the statistical units from scanner peculiarities as it resorts to a relative distance measure. By leveraging the Euclidean distance between lesions to build up the tree representation, hierarchical clustering acts as standardization and normalization strategy. As a consequence, the obtained objects confirm their agnostic nature. Of note, the proposed pipeline displays a modular structure that makes it suitable for different kinds of metastatic or multi-lesion tumors. The dimensionality reduction strategy, distance, and linkage selection in the hierarchical clustering algorithm can indeed be changed and tuned on case study data. Particularly, the proposed approach would be useful in those tumors for which no a priori ordering of lesions is known, for instance in lymphoma. According to the task to be performed, trees can be fed into the classification, clustering, or survival models with the aim of supporting clinical practice in effective treatment planning and monitoring.

The limitations of the present study include the relatively small population analyzed and the consequent use of clinical variables as dichotomous (e.g., Gleason score). Indeed, this study included a sub-group (i.e., patients with at least two lesions identified by [^18^F]FMCH PET/CT) of a larger cohort of recurrent PCa patients prospectively enrolled in an observation trial [13]. Nonetheless, this approach, even if proposed in recurrent PCa patients imaged with [^18^F]FMCH, might be successfully applied to patients affected by metastatic neoplasms or systemic diseases (e.g. lymphoma) imaged with other tracers (e.g., [^18^F]FDG). 

## 5. Conclusions

The proposed approach, developed in PCa patients imaged using [^18^F]FMCH PET/CT, allowed us to clearly represent the coexistence of different radiomic profiles for lesions within each patient and providing insightful information regarding lesion heterogeneity. Collectively, radiomics has brought a rare opportunity for advanced image analysis and it can be used together with artificial intelligence to refine the concept of “personalized medicine”. 

## Figures and Tables

**Table 1:** Baseline patient characteristics.

Variable		Number of Patients (%)
Number of metastases	Oligo (<3)	15 (27%)
	Multi (≥3)	40 (73%)
	Oligo (<5)	26 (47%)
	Multi (≥5)	29 (53%)
	Intermediate (3 ≤ n < 5)	11 (20%)
Gleason Score (dichotomous)	<7	7 (13%)
	=7	24 (44%)
	>7	18 (33%)
	Missing	6 (10%)
Ongoing therapy (ADT)	Y	24 (44%)
	N	31 (56%)
Primary treatment (initial therapy)	RP	15 (27%)
	RP+RT	30 (54%)
	RT	7 (13%)
	Missing	3 (6%)
PSA (dichotomous)	≤1.93 *	11 (20%)
	>1.93	33 (40%)
	Missing	11 (20%)

ADT: androgen deprivation therapy; N: no; PSA: prostate specific antigen; RP: radical prostatectomy; RT: radiation therapy; Y: yes. * Value identified as significant in a larger population [13].

**Table 2:** Clusters of lesions as grouped by mini-batch k-means clustering were characterized in terms of TLA, SUV, site, and GLCM entropy and results are displayed (*p*-values).

Parameter		Cluster 1	Cluster 2	*p*-Value
SUV_max	Median	9.8350	10.8707	0.0187 *
	Std. Dev.	3.9163	7.8156	
	Q3	12.4980	16.7168	
TLA (mL)	Median	4.6851	6.0573	<0.001 ***
	Std. Dev.	14.4136	90.1964	
	Q3	14.1085	65.8136	
GLCM Entropy	Median	1.4224	1.4524	0.0517 .
	Std. Dev.	0.4507	0.6868	
	Q3	1.8524	2.2928	
Organ	Regional lymph nodes	24 (15.4%)	32 (18.1%)	0.4202
	Distant lymph nodes	40 (25.6%)	36 (20.3%)	
	Skeleton	92 (59%)	109 (61.6%)	

GLCM: gray-level co-occurrence matrix; SUV_max: maximum standardized uptake values; Q3: third quartile; Std. Dev.: standard deviation; TLA: total lesion activity. Significance is labelled with *** where *p* < 0.001, with * when *p* < 0.05, and “.” when *p* < 0.1.

**Table 3:** Tests on PSA, GS, oligo/multi-metastatic status, number of lesions, type, and response to therapy highlighted differences among the two patients’ phenotypes, i.e., homogeneous and heterogeneous (*p*-values).

Parameter		Homogeneous	Heterogeneous	*p*-Value
PSA	Median	2.81	3.99	0.3189
	Std. Dev.	1.5036	105.6279	
	Q3	3.81	14.4974	
GS	Median	7.0	7.0	0.7047
	Std. Dev.	0.8314	1.2447	
	Q3	8.0	8.0	
Nodal lesions	Median	2.0	7.0	0.0001 ***
	Std. Dev.	0.8314	3.1359	
	Q3	2.0	10.0	
Total Tumor Volume (mL)	Median	1.9114	15.3200	0.0651 .
	Std. Dev.	5.7938	44.7814	
	Q3	7.0262	31.1022	
Gleason Category	≤7	5 (55%)	26 (65%)	0.5954
	>7	4 (45%)	14 (35%)	
Oligo or Multi (>3)	Oligo	7 (70%)	37 (82%)	<0.0001 ***
	Multi	3 (30%)	8 (18%)	
Oligo or Multi (>5)	Oligo	10 (100%)	29 (64%)	0.0004 **
	Multi	0 (0%)	16 (36%)	
3 < Lesions ≤ 5	<3	7 (70%)	29 (64%)	0.0001 ***
	3 < Lesions ≤ 5	3 (30%)	8 (18%)	
	> 5	0 (0%)	8 (18%)	
Initial Therapy	RP + RT	5 (55%)	25 (58%)	0.6293
	RP	3 (33%)	12 (28%)	
	RT	1 (12%)	6 (14%)	
Ongoing Therapy	N	6 (60%)	25 (55%)	0.7976
	Y	4 (40%)	20 (35%)	
Combined therapy	N	7 (78%)	26 (66%)	0.8810
	Y	2 (22%)	13 (34%)	
Response to therapy	N	9 (100%)	29 (74%)	0.4255
	Y	0 (0%)	10 (26%)	

GS: Gleason Score; N: no; PSA: prostate specific antigen; Q3: third quartile; RP: radical prostatectomy; RT: radiation therapy; Std. Dev.: standard deviation; Y: yes. Significance is labelled with *** where *p* < 0.001, with ** when *p* < 0.01, and “.” when *p* < 0.1.

**Table 4:** Discrimination power of the number of phenotypes, number of lesions, sum of tree branch lengths, and lesions’ radiomic dispersion in stratifying patients according to type of therapy, number of therapies, oligo/multi-metastatic status, Gleason score, PSA, and response to therapy (*p*-values). The number of phenotypes has been computed according to Silhouette coefficient (# phenotypes—silhouette), Calinski-Harabasz index (# phenotypes—CH), and Davies–Bouldin index (# phenotypes—DB).

Parameter	# Phentypes_Silhouette	# Phentypes_ch	# Phentypes_db	Dispersion	Sum Branches
PSA	0.0214	0.0234	0.0210	0.4953	0.0433
GS	0.0736	0.1976	0.3403	0.4672	0.1909
Nodal Lesions	<0.0001	<0.0001	<0.0001	0.0016	<0.0001
Total Tumor Volume	0.0002	<0.0001	0.0004	0.4010	0.0020
Gleason Category	0.0004	0.0006	0.0086	0.0050	0.0061
Oligo or Multi (>3)	0.0002	0.0003	0.0003	0.0008	0.0003
Oligo or Multi (>5)	0.0088	0.0098	0.0119	0.6702	0.2933
3 < Lesions ≤ 5	0.0014	0.0016	0.0020	0.0344	0.0070
Initial Therapy	0.1931	0.2040	0.1908	0.1503	0.1444
Ongoing Therapy	0.6647	0.7010	0.6760	0.7529	0.8379
Combined therapy	0.6245	0.2221	0.5707	0.7968	0.6055
Radiotherapy	0.0003	0.0001	0.0003	0.0207	<0.0001
Hormonotherapy	0.0783	0.6348	0.8975	0.7963	0.0717
Difosfonate	0.1608	0.2336	0.1212	0.1444	0.0727

Abbreviation: GS = Gleason score; PSA = prostate specific antigen.

## Data Availability

The data presented in this study are available in this article and supplementary material.

## Supplementary Materials

The following supporting information can be downloaded at: https://www.mdpi.com/article/10.3390/cancers15030823/s1, Figure S1: Comparison between depth measures: scatterplots of the Mahalanobis, Tukey, Simpli-cial depths on non-standardized data (left panel) compared to standardized data (right panel), Figure S2: Dependency of information provided by the radiomic views, according to ranking agreement analysis (SuperRanker), Figure S3: Lesions’ similarity-based clustering was doublechecked with a ranking aggregation procedure.
