# Supplementary material for: Radiomics-Based Inter-Lesion Relation Network to Describe [18F]FMCH PET/CT Imaging Phenotypes in Prostate Cancer
Source: Cancers (Basel). 2023 Jan 29;15(3):823. doi: 10.3390/cancers15030823 (PMC9913254; doi:10.3390/cancers15030823)
Supplement: Supplementary file 1 [file cancers-15-00823-s001.zip › cancers-2156492-supplementary.pdf]

cancers

MDPI

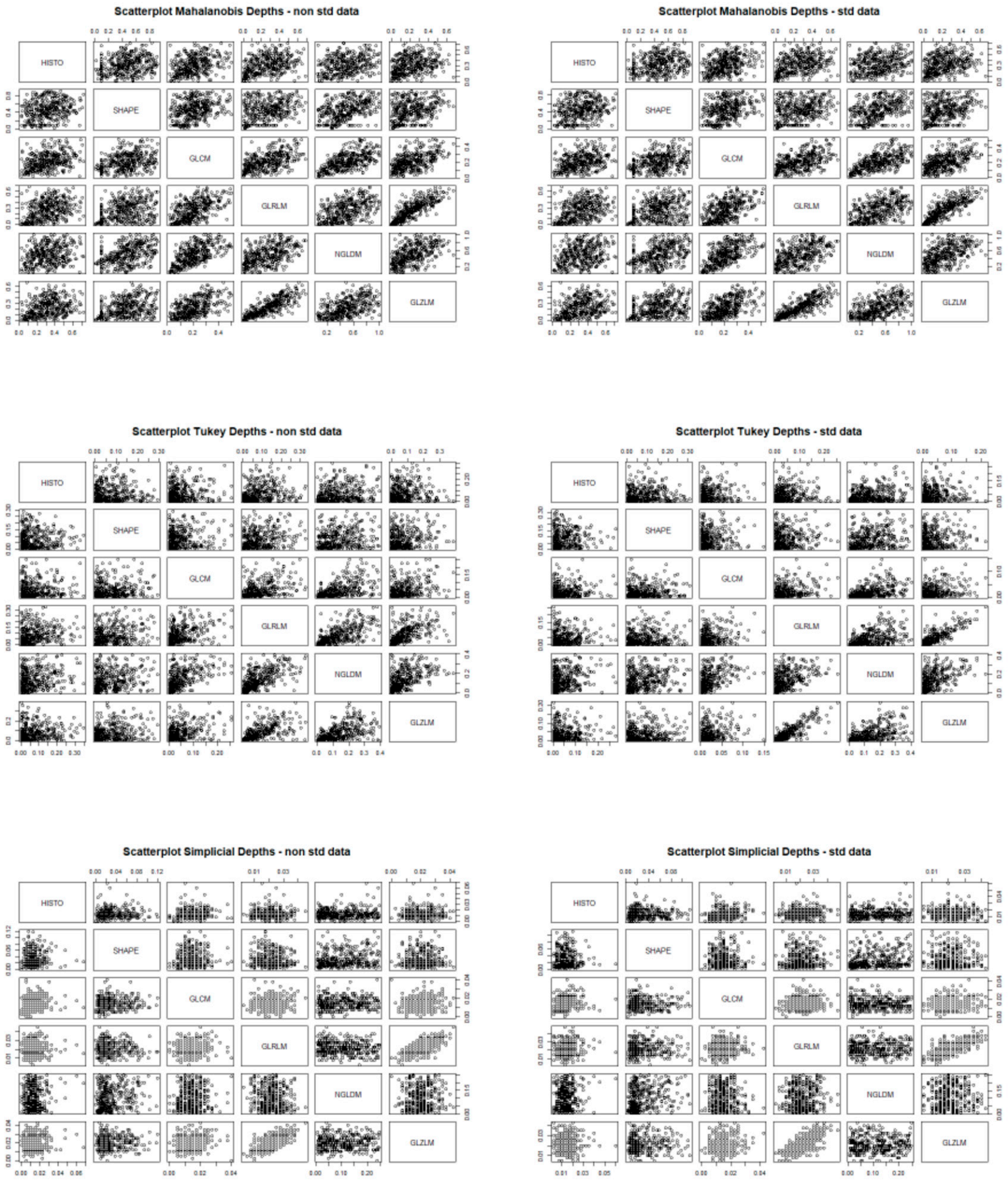

Figure S1. Comparison between depth measures: scatterplots of the Mahalanobis, Tukey, Simplicial depths on non-standardized data (left panel) compared to standardized data (right panel). Of note, Mahalanobis measure provides more dispersed results with respect to Tukey and Simplicial depths, whether they are applied to standardize data or not. Tukey depth values are flatted towards zero, while Simplicial depths assume more discrete values, due to rounding policies. Regardless of the depth definition, radiomic views present very low correlations, with higher values between GLRLM and GLZLM.

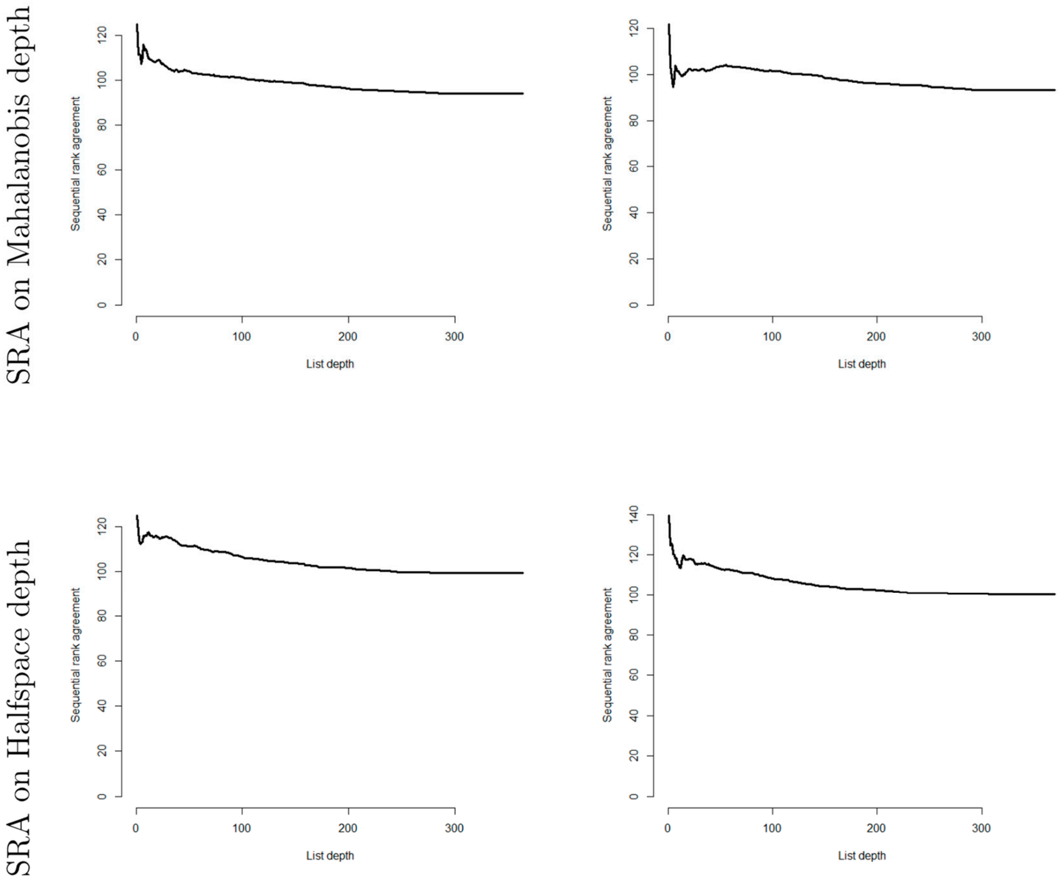

Figure S2. Dependency of information provided by the radiomic views, according to ranking agreement analysis (SuperRanker). The sequential rank agreement (*sra*) methods for comparison of ranked lists was used: the *sra* metrics is the pooled standard deviation of the sets of items ranked less than or equal to a value *d* in any of the ranking lists. The *sra* metrics can be analyzed by letting *d* vary from 0 to the number of elements to compare. Values of *sra* close to zero at any depth *d* suggest that the ranking lists agree while larger values suggest disagreement. In figure, the *sra* rank agreement plot for Mahalanobis and Halfspace depths on both non standardized (left) and standardized data (right) is displayed: in none of the four cases plots are close to zero. This disagreement should not be interpreted as absence of correlation between the radiomic groups, which was proved to be positive; instead, the ranking of lesions induced by view-specific depths is never equal, as expected, suggesting that radiomic groups capture different, yet not independent, aspects of texture description.

	CE-Kendall	GA-Kendall	CE-Spearman	GA-Spearman
Rank 1	Les 3	Les 3	Les 3	Les 3
Rank 2	Les 4	Les 4	Les 4	Les 4
Rank 3	Les 10	Les 10	Les 10	Les 10
Rank 4	Les 5	Les 5	Les 5	Les 5
Rank 5	Les 7	Les 7	Les 8	Les 8
Rank 6	Les 8	Les 8	Les 7	Les 7
Rank 7	Les 1	Les 1	Les 1	Les 1
Rank 8	Les 9	Les 9	Les 9	Les 9
Rank 9	Les 2	Les 2	Les 2	Les 2
Rank 10	Les 6	Les 6	Les 6	Les 6

Figure S3. Lesions' similarity-based clustering was doublechecked with a ranking aggregation procedure. Specifically, depth measures of lesions were transformed into ordered lists as to sort observations from the more central to the furthest. Each lesion was thus described by six positional indexes, one per radiomic group, providing a ranking of observations. The package RankAggreg was used to perform aggregation of the ranking lists using two different aggregating algorithms – Cross-Entropy Monte Carlo (CE) algorithm and Genetic algorithm (GA) – and two types of distances – Spearman footrule and Kendall's tau. The aggregated ranking list provides the consensus among all radiomic views of the lesions' ordering and was used to confirm the similarity between lesions and their clustering in phenotypes. In figure, the radiomic-specific rankings of the n lesions of one sample patient are aggregated in four super ranking lists through the four weighted rank aggregation algorithms: lesions are then ordered from the deepest (Rank 1) to the outer (Rank 10). Colors delimit the three clusters of phenotypes as computed by the clustering similarity measures. Of note, lesions with consecutive ranks are found within the same cluster, enforcing the imaging subtyping policy. The same checking procedure was carried out successfully in each patient.
